# Ketamine sedation during air enema reduction of pediatric intussusception: Assessing safety and intraluminal pressure

**DOI:** 10.1111/ped.15835

**Published:** 2024-11-21

**Authors:** Jun Sung Park, Dahyun Kim, Min Kyo Chun, Jeeho Han, Seung Jun Choi, Jong Seung Lee, Jeong‐Min Ryu, Choong Wook Lee, Pyeong Hwa Kim, Hee Mang Yoon, Young Ah Cho, Jeong‐Yong Lee

**Affiliations:** ^1^ Department of Pediatric Emergency Care Medicine, Asan Medical Center University of Ulsan College of Medicine Seoul Korea; ^2^ Department of Emergency Medicine, Asan Medical Center University of Ulsan College of Medicine Seoul Korea; ^3^ Department of Radiology and Research Institute of Radiology, Asan Medical Center University of Ulsan College of Medicine Seoul Korea

**Keywords:** air enema reduction, intussusception, ketamine, procedural sedation

## Abstract

**Background:**

Recent reports have demonstrated promising results regarding the use of ketamine sedation for reducing pediatric intussusception without an associated elevated risk of bowel perforation. However, data on direct intraluminal pressure are still lacking. This study aimed to investigate sedation safety, primarily by comparing intraluminal pressure.

**Methods:**

This retrospective study included patients aged 10 years or younger, diagnosed with intussusception at a university‐affiliated pediatric emergency department (ED) between July 2021 and February 2023. These patients were categorized into two groups: sedation and non‐sedation. During regular working hours (from 9:00 a.m. to 5:00 p.m. on weekdays), patients were administered 1 mg/kg of intravenous ketamine for sedation during air enema reduction. Patients within non‐working hours did not receive sedative interventions.

**Results:**

In a study of 114 patients with intussusception (median age: 25 months), 29 (25.4%) received sedatives, and 85 (74.6%) did not. Maximum intraluminal pressure during the procedure showed no significant difference between the groups (sedation: 64 mmHg, non‐sedation: 83 mmHg, *p* = 0.091). Bowel perforation was not observed in the overall cohort. No difference was observed in the failure rate or recurrence rate within 24 h between the two groups. Sedation with a median dose of 1 mg/kg ketamine did not cause delays in the ED process and demonstrated no adverse events while maintaining appropriate sedation depth with sequential dosing.

**Conclusions:**

The utilization of ketamine sedation during fluoroscopy‐guided air enema reduction for pediatric intussusception was not associated with increased intraluminal pressure, increased rate of reduction failure, or bowel perforation.

## INTRODUCTION

Intussusception is the predominant cause of intestinal obstruction and abdominal surgery among children younger than 2 years, resulting from the invagination of one intestinal segment into a more distal one.^1^ Fortunately, non‐surgical enema reduction frequently obviates the necessity for surgery,^2^ especially the utilization of fluoroscopy‐guided air enema reduction, a method renowned for its reliability. This technique has a higher success rate of 82.7% (95% confidence interval, CI; 79.9%–85.6%) than that of saline‐enema reduction, which has been reported as 69.6% (95% CI, 65.0–74.1%).^3^ It offers easier confirmation of reduction, minimal chances of perforation‐related complications, easy implementation, and the ability to achieve higher intraluminal bowel pressure.^4^, ^5^

Nevertheless, the majority of air‐enema reductions are performed without sedation, despite the potential distress for young patients.^6^ Several articles concerning the potential risks of sedation during the procedure have frequently cited a study detailing increased colonic perforation and intraluminal pressure in sedated pigs in comparison with their conscious counterparts.^7^ This study demonstrates that sedation can prevent the Valsalva maneuver—a mechanism that diminishes transmural pressure and protects against perforation—resulting in increased intraluminal pressure. Lack of a Valsalva maneuver is one of the well‐known causes of colonic perforation, along with high insufflation pressure (120 mmHg), duration of symptoms for more than 12 hours, dehydration, and younger age.^6^, ^7^

Considering that sedation helps alleviate anxiety and discomfort, promoting a more cooperative and relaxed state in the child, which can minimize any potential movement, resistance, and psychological impact of the procedure, multiple endeavors have been undertaken to administer sedatives during the procedure, demonstrating its safety and efficacy: the success rate ranged from 65.1% to 100%, and perforation rate ranged from 0.3% to 2%.^6^, ^8^, ^9^, ^10^ Recent studies could not find a significant association between sedation during the procedure and intestinal perforation or unsuccessful reduction, thereby challenging the conventional practice of withholding sedation to reduce ileocolic intussusception in children.^6^, ^9^ However, these promising reports focused primarily on comparing bowel‐perforation rates and did not measure intraluminal pressure directly.

In this study we therefore evaluated the safety of sedation during fluoroscopy‐guided air enema reduction in pediatric patients with intussusception by comparing the perforation rate and measuring intraluminal pressure directly using a digital manometer.

## MATERIALS AND METHODS

### Study design and population

We conducted a retrospective study on pediatric patients aged 10 years or younger, diagnosed with intussusception at a tertiary university‐affiliated hospital's emergency department (ED) between July 2021 and February 2023. The ED of the study affiliate evaluates approximately 35,000 pediatric patients annually. Patients with a history of abdominal surgery, perforation on arrival, trapped fluid on ultrasound, or suspected intra‐abdominal anomalies were excluded. Intussusceptions other than the ileo‐cecal (IC) type (e.g., colo‐colic and ileo‐ileal) were also excluded. A recurrence of intussusception within 48 h was regarded as the same case and excluded, whereas a recurrence after 48 h was considered a separate case and included in the analysis.

Patient management and subgroup allocation are as follows: Once intussusception was suspected after using point‐of‐care ultrasound by ED physicians, patients were then transferred to the pediatric radiology department for radiologist‐performed ultrasound (RADUS). When the intussusception was confirmed and the contraindications for air enema reduction (e.g., perforation) were excluded through RADUS, the patients were allocated to either the sedation or non‐sedation group based on their ED visiting time. The sedation group comprised patients visiting during working hours (weekdays from 9:00 a.m. to 5:00 p.m.), whereas the non‐sedation group included those visiting during non‐working hours (weekdays between 5:00 p.m. and 9:00 a.m., weekends, or national holidays) when faculties physicians were limited. Air enema reduction was conducted sequentially. During working hours, reduction was performed by second‐year radiology residents rotating through the division of pediatric radiology, with backup provided by pediatric radiology faculties. During non‐working hours, reduction was performed by third‐year radiology residents rotating through the division of emergency radiology, with backup provided by emergency radiology faculties. The sedation group received sedatives immediately before the procedure, administered by accompanying ED physicians, and intraluminal pressure was controlled to a maximum of 120 mmHg to avoid bowel perforation. Patients who underwent successful air enema reduction for the intussusception were discharged approximately 6 h later and monitored within ED for bowel perforation and recurrence. Procedure failure was defined as cases where intussusception reduction was not achieved despite applying a pressure of over 120 mmHg during air reduction, or where bowel perforation occurred.

The medical records containing detailed demographic and clinical information of the patients, such as age, sex, medical history, laboratory results on‐arrival at ED, sedation record, and hospital course, were thoroughly reviewed. This study was approved by the institutional review board of the Asan Medical Center (approval no. 2023–0348), and the requirement for informed consent was waived due to the retrospective nature of the study.

### Sedation protocol

This study employed standardized definitions outlined in the Quebec Guidelines, a consensus‐based document developed by North American experts in pediatric procedural sedation.^11^ Intravenous (IV) ketamine was selected as the sedative due to its dual effect on sedation and analgesia, ability to induce antegrade amnesia, short half‐life, and rapid onset time.^12^

Once the decision was made for the patient to undergo air reduction with sedation, the ED physician explained the purpose and method of sedation and obtained informed consent. A pre‐sedative assessment was conducted, which included the patient's vital signs, oxygen saturation, weight, age, dental condition, signs of upper airway obstruction, and American Society of Anesthesiologists physical status.^13^ The patient was then transferred for a confirmative RADUS and fluoroscopy, equipped with a portable oxygen tank, oxygen supply tools, and airway devices. The ED physician monitored oxygen saturation and electrocardiograms continuously during the patient's transfer and until the patient's return to the ED. An initial IV dose of 1 mg/kg of ketamine was administered just before the procedure, once all examiners were prepared and the patient was positioned on the fluoroscopy table. We aimed to maintain the sedation depth around level three on the Pediatric Sedation State Scale (PSSS), with additional doses of 1 mg/kg as needed.^14^ Any adverse events,^11^ sedation depth, vital signs, and dosage of sedatives were recorded by the ED physician. All ED physicians involved in monitoring the sedation process underwent a monthly sedation training course in the pediatric ED.

### Intraluminal pressure measuring

**FIGURE 1:**
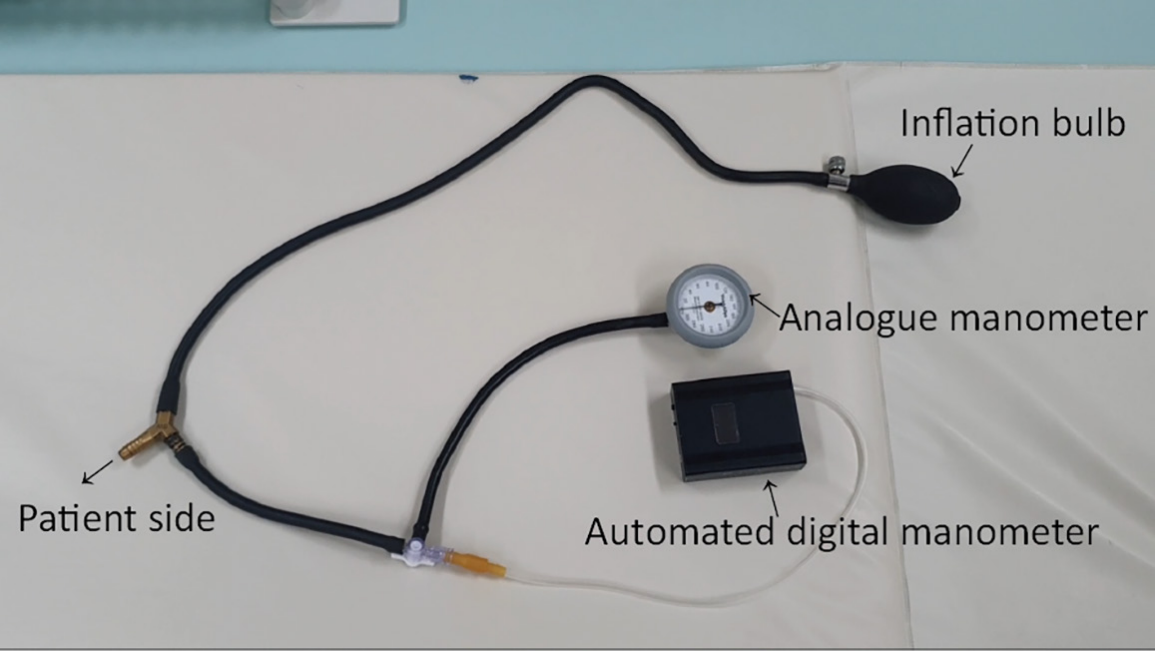
The digital manometer used in the study.

**FIGURE 2:**
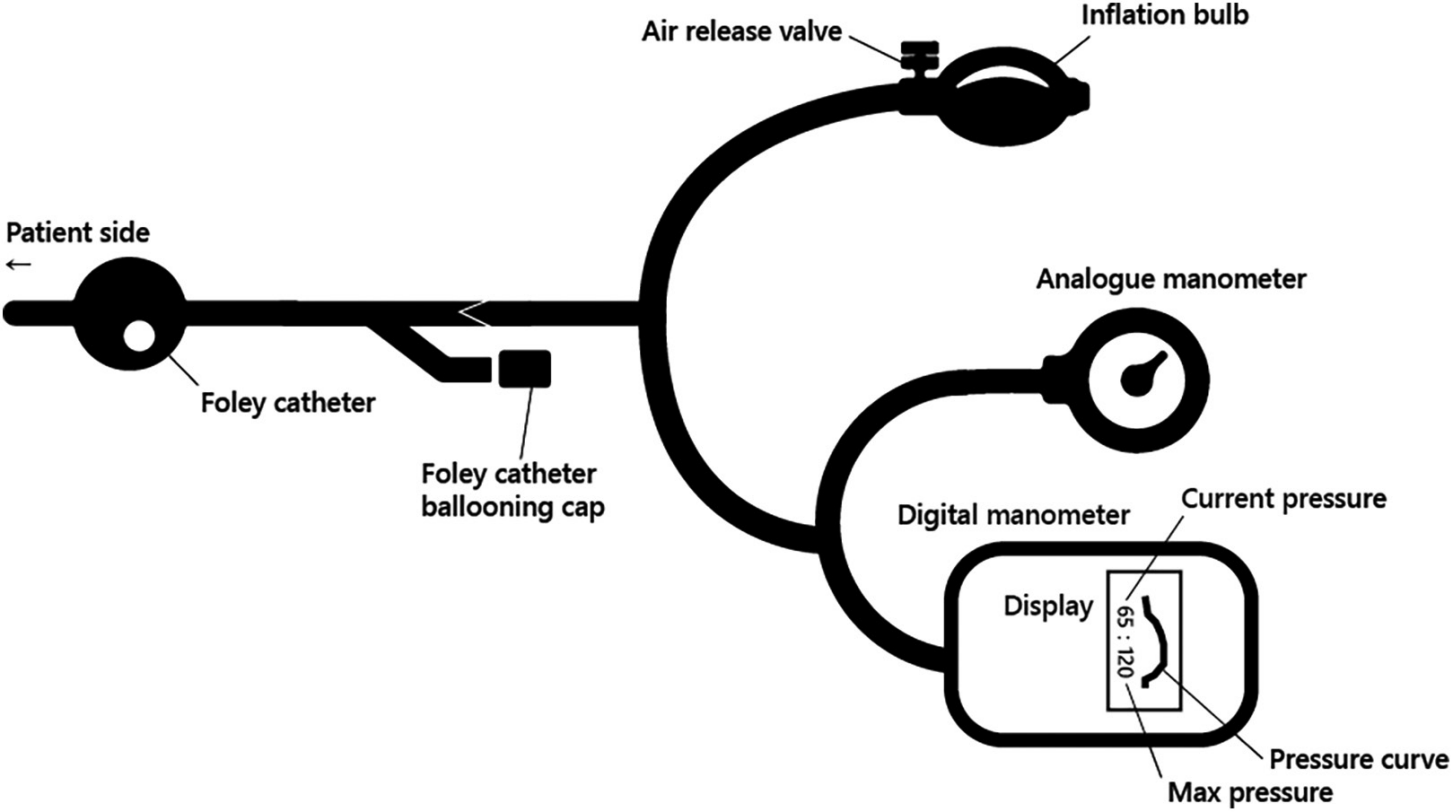
Schemed illustration of digital manometer used in the study.

Potential biases arising from operator recall while visually assessing the actively moving probe on the manometer were mitigated through the utilization of an in‐house automatic digital manometer to ensure accurate measurement of the maximum pressure during the procedure (Figures 1 and 2). This device promptly nullified any transient disequilibrated pressure caused by the pumping of the rubber bulb and provided real‐time current pressure as well as the maximum pressure throughout the procedure on the display (Supporting Information, Video S1). The pressure profile could subsequently be reviewed on the manometer's monitor after the reduction procedure. Furthermore, the maximum pressure attained during the reduction was also documented by the operator through an analog manometer, which was integrated with the compressor tube of the digital manometer. The maximum pressure measured by the digital manometer was adopted after being reviewed by the operator, considering the pressure co‐measured by analog manometer. No patient rejected the pressure measurement using a digital manometer.

### Outcomes

The primary outcome aimed at comparing the maximum pressure, failure rate, and bowel perforation rate observed during the air reduction procedure based on the administration of sedation. The secondary outcomes included the occurrence of adverse events^11^ associated with sedatives and comparative analysis of the ED workflow (e.g., door‐to‐reduction time, ED length of stay) when sedation was utilized.

### Statistical analysis

To compare the groups, the Mann–Whitney *U*‐test was utilized for continuous variables with a non‐normal distribution, whereas the Fisher's exact test and a *χ* ^2^ test were used for categorical variables, as appropriate. These analyses were performed using SPSS Statistics for Windows, version 21.0 (IBM Corp., New York, NY, USA). A *p* value of <0 .05 was regarded as statistically significant.

## RESULTS

**FIGURE 3:**
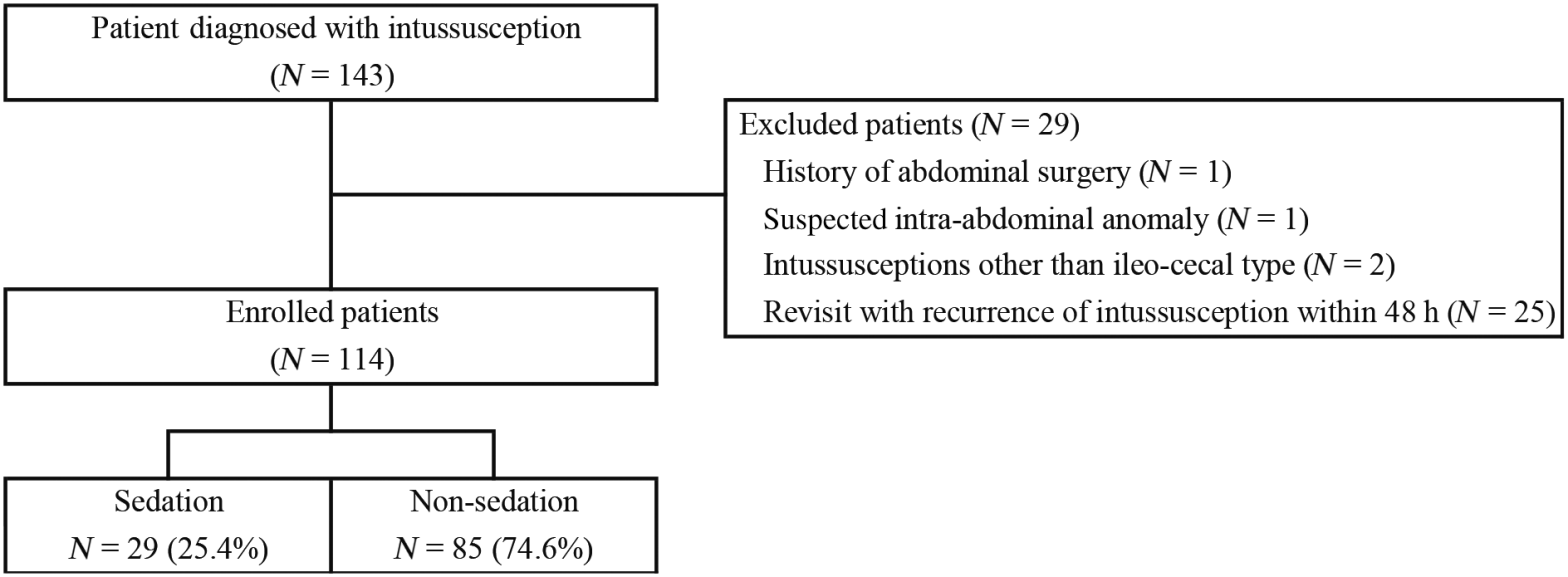
Flowchart of the study population.

A total of 143 patients were diagnosed with IC‐type intussusception during the study period (Figure 3). After exclusions, 114 patients were included in the final analysis. During the working time, 29 (25.4%) patients visited, and they were classified into the sedation group. The other 85 (74.6%) patients were classified into the non‐sedation group (Table 1). No difference in terms of demographics and clinical presentations was observed between the groups. The laboratory test results did not differ between the groups.

**TABLE 1:** Demographic and clinical characteristics of the study.

	Sedation	Non–sedation	Total	*p*
	*N* = 29 (25.4%)	*N* = 85 (74.6%)	*N* = 114	
Age, month	28 (24 to 44)	24 (14.5 to 37)	25 (17 to 39)	0.098
Sex, male	19 (65.5)	59 (69.4)	78 (68.4)	0.697
Clinical presentation				
Duration of symptom, h	6 (2 to 24)	5 (1.75 to 12)	5 (2 to 12)	0.125
Abdominal pain	24 (82.8)	65 (76.5)	89 (78.1)	0.480
Vomiting	9 (31)	39 (45.9)	48 (42.1)	0.162
Hematochezia	2 (6.9)	10 (11.8)	12 (10.5)	0.728
Past history of intussusception	5 (17.2)	10 (11.8)	15 (13.2)	0.451
Pathologic leading point	1 (3.4)	1 (1.2)	2 (1.8)	—
Laboratory test				
WBC, ×10^3^/μL	10.4 (9.2 to 13.9)	12.2 (9.0 to 14.8)	11.5 (9.2 to 14.6)	0.299
CRP, mg/dL	0.26 (0.1 to 1)	0.18 (0.1 to 0.7)	0.2 (0.1 to 0.72)	0.497
Glucose, mg/dL	93 (82 to 107)	102 (87 to 113)	100 (86 to 111)	0.186
pH	7.41 (7.38 to 7.46)	7.41 (7.36 to 7.44)	7.41 (7.37 to 7.44)	0.277
Bicarbonate, mmol/L	20.6 (17.5 to 21.9)	20.1 (18.1 to 21.2)	20.2 (18.3 to 21.3)	0.609
BE, mmol/L	−3 (−5.1 to −1.7)	−4 (−5.5 to −2.6)	−4 (−5.5 to −2.5)	0.108
Lactate, mmol/L	1.6 (1.2 to 2)	1.6 (1.3 to 2.1)	1.6 (1.3 to 2.1)	0.789

*Note*: Values are presented as numbers (%) or medians (interquartile range).

Abbreviations: BE, Base excess; CRP, C‐reactive protein.

The maximum intraluminal pressure of the overall cohort was 80 (interquartile range, 60–103) mmHg (Table 2). There was no statistically significant difference in the maximum intraluminal pressure between two groups (median, 64 vs. 83 mmHg, *p* = 0.091). There was also no statistically significant difference in the failure rate of reduction (3.4% vs. 18.8%, *p* = 0.067) and recurrence rate within 24 h between the groups (17.2% vs. 15.3%, *p* = 0.824). No bowel perforation was observed across the overall cohort. There was also no delay in the ED workflow process, including door‐to‐reduction time (median, 110 vs. 128 min; *p* = 0.124).

**TABLE 2:** Clinical course of the patients.

	Sedation	Non–sedation	Total	*p*
	*N* = 29 (25.4%)	*N* = 85 (74.6%)	*N* = 114	
Maximum pressure, mmHg	64 (59–99)	83 (60–115)	80 (60–103)	0.091
Reduction fail on the first attempt	1 (3.4)	16 (18.8)	17 (14.9)	0.067
Recurrence of intussusception in 24 h	5 (17.2)	13 (15.3)	18 (15.8)	0.824
Bowel perforation	—	—	—	—
Door‐to‐reduction time, min	110 (64–171)	128 (102–164)	125 (92–166)	0.124

*Note*: Values are presented as number (%) or median (interquartile range).

Most patients with sedation were administered only an initial dose of IV ketamine (1 mg/kg) during the procedure, except for two patients (patient numbers 1 and 2) (Supporting Information, Table S1). The median sedation depth was three (i.e., expression of pain or anxiety on face without moving or impeding completion of the procedure, no requirement for restraint). No sedation‐related adverse event was observed.

## DISCUSSION

In our study, we demonstrated the safety of sedation in fluoroscopy‐guided air enema reduction for pediatric intussusception by measuring the intraluminal pressure directly, using a digital manometer, and maintaining a proper sedation protocol. Despite the small sample size of the sedation group, we observed only one case of reduction failure and no case with bowel perforation. Moreover, there was no statistically significant delay to fluoroscopy‐guided air enema reduction with or without of sedation. An initial dose of 1 mg/kg of IV ketamine, with additional doses as required, maintained appropriate sedation depth without causing adverse events. These findings could contribute as evidence to the recent reports on the safety and efficacy of procedural sedation and anesthesia (PSA) in reducing pediatric intussusception.^6^, ^8^, ^10^, ^15^, ^16^, ^17^, ^18^

In the context of using PSA during intussusception reduction, various methods, sedative types, and dosages have been employed.^6^, ^8^, ^10^, ^15^, ^16^, ^17^ Studies have shown no difference or even an increase in success rates, and no difference in perforation risk.^10^ However, recent large‐scale cross‐sectional study^6^ and systematic reviews^17^, ^19^ still cite the experimental animal study by Shiels et al.^7^ conducted in 1993, which investigated the increased risk of perforation associated with sedatives. In that study, pigs were allocated into two groups: a deep sedation group, in which the pigs were anesthetized fully by administering both halothane gas and 22 mg/kg of intramuscular (IM) ketamine to eliminate the ability to perform the Valsalva maneuver, and the light sedation group, in which they were administered only 22 mg/kg of IM ketamine to enable the Valsalva maneuver. The study concluded that deep sedation led to a loss of the Valsalva maneuver, resulting in higher intraluminal pressure in comparison with the shallow sedation group (121 vs. 145 mmHg). In our study, we used low‐dose ketamine to maintain a sedation depth of around 3, which likely allowed for the Valsalva maneuver. Consequently, the maximum intraluminal pressure observed in the sedation group did not exceed that of the non‐sedation group. Proper sedation depth allowing the Valsalva maneuver might therefore be key to safe PSA during the procedure. Our study does not directly contradict the findings of Shiels et al., but rather suggests that maintaining an appropriate sedation depth can allow for successful reduction without an increase in intraluminal pressure. This study can therefore serve as a preliminary study for validating the safety and efficacy of PSA in intussusception reduction through further larger‐scale prospective research.

We chose IV ketamine as a sedative agent because ketamine provides potent analgesia, sedation, and amnesia while maintaining cardiovascular stability and preserving spontaneous respirations and airway reflexes.^20^ Severe adverse events are rare (<0.5%) during pediatric procedural sedation with ketamine in the ED.^21^ In particular, a lower dose of 1.6 mg/kg on average rarely cause adverse events.^21^ Unlike the 2 mg/kg often used as an initial dose for many procedural anesthetics, we administered an initial dose of 1 mg/kg of ketamine, supplemented by an additional dose of 1 mg/kg as required to maintain the appropriate sedation depth.^22^, ^23^ Furthermore, as the sedation depth should be quickly evaluated in real time during the procedure by the ED physician, we used PSSS as an assessment scale due to its simplicity and intuitivity.^14^

Approximately two‐thirds of the patients were still not administered proper PSA during the procedure.^6^ As the environment of ED itself could be a source of fear and anxiety for pediatric patients, effectively managing such fear, anxiety, and pain is a key factor for the wellbeing of children seeking treatment in ED.^24^ Intussusception, in particular, causes cyclic abdominal pain, anxiety, and fear, necessitating PSA. In such a fearful state, applying colonic pressure through the anus maximizes pain and fear in the children and prevents them from cooperating, which can impede the procedure and cause psychogenic trauma. Whenever feasible, therefore, creating a calm environment for patients, guardians, and operators through appropriate sedation is imperative. The importance of antegrade amnesia to prevent mental trauma is more greater in comparison with other conditions requiring painful procedures, due to the potential recurrence of intussusception and the likelihood that patients will need to undergo the same procedure repeatedly. The use of sedatives during intussusception air reduction therefore needs to be validated and implemented through future studies.

The major limitation of the study is small sample size in the sedation group derived from the study design based on non‐randomized allocation. Although there were no cases of bowel perforation in the overall cohort, the small sample size makes it difficult to compare the risk between the groups. We had to implement PSA exclusively during working hours, which accounted for 23.8% of the week (40 h out of 168 h), to ensure the availability of attending physicians from the ED, radiology, and pediatric critical care. This decision was driven from the absence of conclusive evidence indicating that sedation might not elevate intraluminal pressure, in contrast to comparative research conducted on pigs. The non‐sedation group showed higher intraluminal pressure, although the difference was not statistically significant. This group, who visited during non‐working hours had more experienced operators in comparison with the sedation group, indicating that operator experience did not impact outcomes significantly. However, the impact of differences in backup attendings and other setup factors between the two groups, although small, cannot be completely excluded. This study also did not investigate whether the Valsalva maneuver, which was identified as a cause of increased intraluminal pressure in a previous study, occurred in each group, nor how it affected the intraluminal pressure. Nevertheless, our study could serve as a preliminary step for Nevertheless, our study could serve as a preliminary step towards a larger, prospective, randomized study specifically designed to measure intraluminal pressure and the effects of the Valsalva maneuver specifically designed to measure intraluminal pressure and the Valsalva maneuver. The single‐centered and retrospective nature of this study means that caution is needed when generalizing the result in other EDs, considering selection bias.

## CONCLUSION

Ketamine sedation during fluoroscopy‐guided air enema reduction for pediatric intussusception was not associated with increased intraluminal pressure, reduction failure rates, or bowel perforation. The use of sedatives during intussusception air reduction therefore needs to be validated and implemented through future studies.

## AUTHOR CONTRIBUTIONS

Jeong‐Yong Lee conceived and designed the study. All authors conducted data collection and analysis. Jun Sung Park, Pyeong Hwa Kim, Hee Mang Yoon and Jeong‐Yong Lee contributed to data interpretation. Jun Sung Park drafted the article. All authors contributed to critical revision and approved the final version.

## FUNDING INFORMATION

This study did not receive any funding.

## CONFLICT OF INTEREST STATEMENT

The authors declare no conflict of interest.

## Supporting information


Appendix S1.
