# Supplementary material for: Ketamine sedation during air enema reduction of pediatric intussusception: Assessing safety and intraluminal pressure
Source: Pediatr Int. 2024 Nov 21;66(1):e15835. doi: 10.1111/ped.15835 (PMC11580367; doi:10.1111/ped.15835)
Supplement: Supplementary file 1 — Appendix S1. [file PED-66-e15835-s001.docx]

| Table S1. Sedation parameters of the patients | | | | | | | | | | | | | | | | | | | | | | | | | | | | | |
| --- | --- | --- | --- | --- | --- | --- | --- | --- | --- | --- | --- | --- | --- | --- | --- | --- | --- | --- | --- | --- | --- | --- | --- | --- | --- | --- | --- | --- | --- |
| Patient No. | 1 | 2 | 3 | 4 | 5 | 6 | 7 | 8 | 9 | 10 | 11 | 12 | 13 | 14 | 15 | 16 | 17 | 18 | 19 | 20 | 21 | 22 | 23 | 24 | 25 | 26 | 27 | 28 | 29 |
| Dosage of ketamine, mg/kg | 1.5 | 2 | 1 | 1 | 1 | 1 | 1 | 1 | 1 | 1 | 1 | 1 | 1 | 1 | 1 | 1 | 2 | 1 | 1 | 1 | 1 | 1 | 1 | 1 | 1 | 1 | 1 | 1 | 1 |
| ASA classification | 2 | 2 | 1 | 2 | 1 | 2 | 2 | 2 | 2 | 2 | 2 | 2 | 2 | 2 | 2 | 2 | 2 | 2 | 2 | 2 | 2 | 2 | 2 | 2 | 2 | 2 | 2 | 2 | 2 |
| Sedation depth | 3 | 3 | 3 | 3 | 4 | 3 | 3 | 3 | 2 | 4 | 3 | 4 | 3 | 3 | 2 | 2 | 3 | 3 | 3 | 2 | 3 | 2 | 3 | 2 | 2 | 2 | 2 | 2 | 2 |
| Adverse event | - | - | - | - | - | - | - | - | - | - | - | - | - | - | - | - | - | - | - | - | - | - | - | - | - | - | - | - | - |
| maximum pressure, mmHg | 50 | 120 | 60 | 60 | 100 | 84 | 64 | 100 | 71 | 64 | 113 | 70 | 80 | 50 | 47 | 40 | 100 | 120 | 83 | 61 | 60 | 60 | 81 | 121 | 19 | 51 | 120 | 64 | 60 |
| Recurrence of intussusception in 24 hours | + | - | - | - | - | - | - | - | - | - | - | - | - | - | + | + | - |  | - | - | - | - | - | - | - | - | + | + | - |
| Reduction fails on the first attempt | - | - | - | - | - | - | - | - | - | - | - | - | - | - | - | - | - | + | - | - | - | - | - | - | - | - | - | - | - |
| Bowel perforation | - | - | - | - | - | - | - | - | - | - | - | - | - | - | - | - | - | - | - | - | - | - | - | - | - | - | - | - | - |
| ASA, American Society of Anesthesiologists. | | | | | | | | | | | | | | | | | | | | | | | | | | | | | |
